# Robust Huber-Based Iterated Divided Difference Filtering with Application to Cooperative Localization of Autonomous Underwater Vehicles

**DOI:** 10.3390/s141224523

**Published:** 2014-12-19

**Authors:** Wei Gao, Yalong Liu, Bo Xu

**Affiliations:** College of Automation, Harbin Engineering University, Harbin 150001, China; E-Mails: gaow@hrbeu.edu.cn (W.G.); xubocarter@sina.com (B.X.)

**Keywords:** robustness, nonlinear state estimation, Huber-based iterated divided difference filtering, autonomous underwater vehicles, cooperative localization

## Abstract

A new algorithm called Huber-based iterated divided difference filtering (HIDDF) is derived and applied to cooperative localization of autonomous underwater vehicles (AUVs) supported by a single surface leader. The position states are estimated using acoustic range measurements relative to the leader, in which some disadvantages such as weak observability, large initial error and contaminated measurements with outliers are inherent. By integrating both merits of iterated divided difference filtering (IDDF) and Huber's M-estimation methodology, the new filtering method could not only achieve more accurate estimation and faster convergence contrast to standard divided difference filtering (DDF) in conditions of weak observability and large initial error, but also exhibit robustness with respect to outlier measurements, for which the standard IDDF would exhibit severe degradation in estimation accuracy. The correctness as well as validity of the algorithm is demonstrated through experiment results.

## 1. Introduction

Localization of Autonomous Underwater Vehicles (AUVs) has always been an attractive problem because localization is acknowledged as an essential capability for an AUV. Due to strong attenuation of electro-magnetic underwater, the navigation of AUVs is usually based on inertial navigation systems (INS). Although the INS is very suitable for AUV navigation, the unbounded increase in error over time goes against the AUV staying submerged for longer operations, even with Doppler aiding. To solve this problem, position fixes are needed to inhibit error increase. The GPS signal can be received near surface; however, it is impractical for AUVs' frequent surfacing when deep-water missions performed. An acoustic baseline system, such as the ultra-short baseline navigation (USBL) and long baseline navigation (LBL), can realize bounded-error position and has been widely and effectively used on many different types of AUVs [1–3]. However, they inevitably suffer from a costly installation or restricted operation area due to static beacons being pre-deployed, *etc.* [4]. In addition, the complicated hardware design, high energy consumption and higher payload demand, which derive from the conventional underwater localization systems, make it unsuitable for the small and simple AUV adoption [5]. In order to increase the autonomy of the vehicle and avoid costly pre-deployment of underwater transponders, another favorable alternative, namely that of terrain-aided navigation or simply terrain navigation, which use observable physical features to obtain an estimate of the AUV's position, has also been proposed in [6–8]. However, underwater maps of the whole area over which the AUV operates must be available. Therefore, a new cooperative localization scheme based on acoustic range measurement has been studied in recent years [9–12]. In this scheme, only a small number of AUVs in the team are equipped with accurate inertial sensors. With the AUVs communicating with each other through acoustic modems on board, the AUVs with high navigational performance will support the others with a low-cost, low precision dead-reckoning (DR) system to bound the localization errors. The cooperative solution does not only allow AUVs to operate with high navigational precision, but also provides low cost and unlimited use of their operating area [4].

As a classical nonlinear filtering algorithm, the extended Kalman filter (EKF) [13] has been widely used to recursively estimate the position of AUVs [4,12,14–16]. Performance comparisons of particle filtering (PF), non-linear least-squares optimization (NLS) solutions and the EKF were proposed in [12], although the post-processed NLS solution achieved best performance, it would not have been available online to the AUVs for motion planning. As for the PF, it is considered to be less suitable for the AUVs' cooperative localization because the large particle cluster is needed to adequately sample the large area of uncertainty [11]. A decentralized extended information filter (DEIF) is designed for AUVs' cooperative navigation in [17]. Although it achieves excellent theoretical results, a real-time implementation requires additional overhead to address the problem of packet loss. A centralized extended Kalman filter (CEKF) is provided in [4], which reports a principled, general approach to tracking the problem of correlation and time delays for AUV navigation based on a single moving reference beacon. However, it relies on concurrent access to the sensor measurements and thus is only applicable in post-processing. Although the EKF has been widely used for AUVs' cooperative localization, it has several deficiencies such as unavoidable large errors or a divergent estimate when the nonlinearities become severe, because the EKF is based upon the principle of linearizing the nonlinear system models via first order Taylor series expansions [18].

The unscented Kalman filter (UKF) [13] and divided difference filter (DDF) [19], known as sigma-point Kalman filter (SPKF), is an efficient derivative free state estimator, which does not linearize the dynamic system for the propagation; instead, it propagates a cluster of points centered on the current estimate to obtain improved approximations of the conditional mean and covariance. In contrast to the basic Kalman filter, the alternative method can easily be extended to determine second-order solutions to the minimum *l*_2_ − norm filtering problem and increases the estimation accuracy when system and measurement equations are nonlinear. The performance of the UKF and DDF is nearly the same; however, because DDF can guarantee a positive semi-definiteness of the posterior covariance matrix, it can obtain a more accurate covariance matrix than the UKF [18]. Therefore, in this work, we make use of the DDF for the AUVs' cooperative localization. Although the DDF outperformed EKF in a nonlinear filtering problem, the standard DDF also shows its weaknesses in robustness, convergence speed and tracking accuracy when the system is of weak observability and large initial error [18], which is inescapable for our work in AUV cooperative localization [12]. For these reasons, the iterated filtering algorithm is usually used to improve the performance of nonlinear filtering algorithm [20–25], which is developed under the general filtering framework and makes full use of the observation information by using an iterative measurement update, so the truncated error of high order can be avoided. Furthermore, the weak observability and large initial error can be satisfied to a certain extent by using the up-to-date estimated information as the initial estimate for each iteration process [22,25]. In this work, therefore, we applied an iterated divided difference filter (IDDF) to the AUV position estimate.

Unfortunately, as indicated in [22], the iterated filtering method is very sensitive to measurement errors. That is to say the *posteriori* update state should be more approximate to the truth than the predicted one. Otherwise there is likely no improved performance obtained, and rather a greatly degraded performance. Similar to the basic Kalman filter, the simplified DDF is also a state estimator that minimizes the *l*_2_ − norm of the residuals and is the maximum likelihood technique assuming that the error statistics follow Gaussian probability distributions [26]. Thus, if the distribution of the true noise deviates from the assumed Gaussian model, characterized by heavier tails and outliers [27], the performance of the DDF can be severely degraded, and then the iterated filtering performance will become worse and worse, because of the iterated using of measurements. This is especially a problem for AUV cooperative localization based on acoustic range observed, in which the acoustic range measurements are of then characterized by contaminated Gaussian noise with outliers due to variable sound speed, ray-bending and multi-paths [4,10]. Therefore, it is imperative to develop an iterated filtering technique that is robust to the outlier measurements for AUV cooperative localization. One such technique is Huber's generalized maximum likelihood estimation theory [28,29], which is a combination of minimum *l*_1_ − and *l*_2_ −norm estimator and has been successfully used for relative navigation filter design for robust rendezvous in lunar orbit and tracking problems [30–32]. The Huber estimator is a combination of the two estimators that seeks to use the best of both techniques, in particular, the robustness of the sample median and the efficiency of the sample mean [30].

In this paper, a novel robust Huber-based iterated divided difference filter (HIDDF) algorithm is proposed, which integrates both merits of IDDF and Huber's M-estimation methodology. By minimizing the Huber objective function, the HIDDF method can improve the robustness to contaminated measurements with outliers in the process of iterated measurement update. The main contribution of this paper includes two parts: (1) by applying the iterated filtering method to AUV cooperative localization, we can achieve a more accurate position estimate in weak observability and faster convergence speed in large initial error; and (2) by combining the iterated method and the Huber's M-estimation methodology, the HIDDF method is robust to the outlier measurements and the stability of the iterated filtering method can be guaranteed. The remainder of this paper is organized as follows. In Section 2, the system model for AUV cooperative localization is described. Section 3 proposes the development of the robust Huber-based iterated divided difference filter algorithm. In Section 4, the simulation results was presented to verify the feasibility and performance of the proposed filtering algorithm, while Section 5 describes and reports results from the actual lake-water field trials. Finally, the conclusion is drawn in Section 6.

## 2. Cooperative Localization with a Single Leader

In this cooperative localization system, only a single surface leader is used as in previous works [12,14], which serves as the communication and navigation aid (CNA). Some powerful and precise sensors are equipped on the CNA to make sure that the reference position can be provided for the submerged AUVs. The accurate position of CNA can be directly obtained by GPS when surfacing or estimated by high-precision integrated navigation system based on inertial navigation system (INS), Doppler velocity log (DVL) and pressure sensor when submerged. Meanwhile, the underwater AUVs are normally equipped themselves with low cost DR and pressure sensors. In order to cooperate during their mission, each AUV equipped itself with an acoustic modem. By communicating with the CNA through the acoustic modems on-board, the AUVs can acquire the referenced position of the CNA and the distances to it and then use them to bound the localization errors accumulated by DR [33].

### 2.1. Dynamic System Model

As indicated in [12,34], because the depth of AUV can be measured precisely through the pressure sensor, the 3-D problem can be simplified to 2-D and the AUV position **x***_k_* = [*x_k_*, *y_k_*, *θ_k_*]*^T^* is propagated by the equations below.

(1) $$\begin{array}{l} x_{k}=x_{k-1}+\Delta t\left({\hat{v}}_{k}\cos{\hat{\theta }}_{k}+{\hat{w}}_{k}\sin{\hat{\theta }}_{k}\right),\\ y_{k}=y_{k-1}+\Delta t\left({\hat{v}}_{k}\sin{\hat{\theta }}_{k}-{\hat{w}}_{k}\cos{\hat{\theta }}_{k}\right),\\ \theta_{k}={\hat{\theta }}_{k}. \end{array}$$

where *θ̂_k_* is the heading measurement, *v̂_k_* and *ŵ_k_* are respectively the forward and starboard velocity measured by DVL and Δ*t* is the sampling period.

According to the dynamical system modem given in Equation (1), the discrete system equivalent is given by

(2) $${\text{x}}_{k+1}=f\left({\text{x}}_{k},\mu_{k}\right)+{\text{w}}_{k}$$

where **μ***_k_* is the control input and it is assumed to be affected by an additive Gaussian noise **w***_k_* ∼ *N* (0, **Q***_k_*).

(3) $$Q_{k}=E\left[{\text{w}}_{k}{\text{w}}_{k}^{T}\right]=\left[\begin{array}{ccc} \sigma_{v_{k}}^{2} & 0 & 0\\ 0 & \sigma_{w_{k}}^{2} & 0\\ 0 & 0 & \sigma_{\theta_{k}}^{2} \end{array}\right]$$

We denote **x̂***_k_* as the position estimate at time step *k*. We estimate the position at time step *k* + 1 *via* the prediction model in Equation (2) to give us **x̄***_k_*_+1|_*_k_*. When the referenced position of surface beacon and acoustic range acquired successfully at time step *k* + 1, we combine the measurement with the estimate **x̄***_k_*_+1|_*_k_* to give us **x̂***_k_*_+1_. Due to the low frequency of acoustic communication (at least 5 s are needed for an acoustic range measurement for a one-way ranging system and at least 10 s for round-trip ranging system) [12], when there is no referenced information received, the system belief should be updated with noise covariance **Q***_k_* according to the performance of the navigation sensor used in each step of AUV state prediction.

### 2.2. Measurement Model

As mentioned above, the CNA broadcasts its referenced position to AUVs periodically *via* acoustic modem, and then the relative range between surface beacon and AUVs can be obtained by means of Time of Arrival. Denote the referenced position of CNA at time *k* as $x_{k}^{r}={\left[x_{k}^{r},y_{k}^{r},z_{k}^{r}\right]}^{T}$, the range measurement function is given by

(4) $${\text{y}}_{k}=g\left({\text{x}}_{k}\right)+{\text{v}}_{k}=\sqrt{{\left(x_{k}^{r}-x_{k}\right)}^{2}+{\left(y_{k}^{r}-y_{k}\right)}^{2}+{\left(z_{k}^{r}-z_{k}\right)}^{2}}+{\text{v}}_{k}$$

where **v***_k_* is the range measurement noise, we denote **H***_k_* as the Jacobin matrix of *g*(**x***_k_*) with respect to **x***_k_* and **R***_k_* as the variance of range measurement.

Observability is an important property for the states' estimate, especially for our work in AUV cooperative localization. In this scheme, only a single surface leader was used; only range information can be observed and low update frequency for cooperative localization. All the cooperative conditions above will result in weak observability for an AUV state estimate; this has been acknowledged and proven by many early studies [5,12,14,35,36]. Although the performance can be improved by using the observed information iteratively, the outlier measurements will cause the estimate divergent. The robustness of the iterated method to the outlier noise is therefore very important.

## 3. Development of Robust Huber-Based Iterated Divided Difference Filter

### 3.1. Divided Difference Filter

This section gives a brief summary of the DDF. The interested reader is referred to Standard DDF, that has been described in [20,35], which linearizes nonlinear functions with Stirling's polynomial interpolation with no differential operations.

Considering the discrete-time nonlinear dynamic system mentioned above:

(5) $$\begin{array}{c} {\text{x}}_{k+1}=f\left({\text{x}}_{k},{\text{u}}_{k}\right)+{\text{w}}_{k}\\ {\text{y}}_{k}=g\left({\text{x}}_{k}\right)+{\text{v}}_{k} \end{array}$$

The DDF algorithm can be summarized as follows. First, the square root decompositions of the predicted state error covariance **P̄***_k_*, corrected state error covariance **P̂***_k_*, process noise covariance **Q***_k_* and measurement noise covariance **R***_k_* are introduced as

(6) $${\hat{P}}_{k}={\hat{\text{S}}}_{\text{x}}{\hat{\text{S}}}_{\text{x}}^{T},{\bar{P}}_{k}={\bar{\text{S}}}_{\text{x}}{\bar{\text{S}}}_{\text{x}}^{T},{\text{Q}}_{k}=S_{\text{w}}S_{\text{w}}^{T},{\text{R}}_{k}=S_{\text{v}}S_{\text{v}}^{T}$$

The factorization of the noise covariance matrices can usually be made in advance. **Ŝ**_x_ and **S̄**_x_ are updated directly during the application of the filter. Let the *j* th column of **S̄**_x_ be denoted as **S̄**_x,j_ and let this also be the case for the other factors.

Then four matrices consisting of divided differences are defined as

(7) $${\text{S}}_{\text{x}\hat{x},k}^{\left(1\right)}=\frac{1}{2h}\left\{{\text{f}}_{i}\left({\hat{x}}_{k}+h{\hat{s}}_{x,j},{\text{u}}_{k},{\bar{w}}_{k}\right)-{\text{f}}_{i}\left({\hat{x}}_{k}-h{\hat{s}}_{x,j},{\text{u}}_{k},{\bar{w}}_{k}\right)\right\}$$

(8) $${\text{S}}_{\text{xw},k}^{\left(1\right)}=\frac{1}{2h}\left\{{\text{f}}_{i}\left({\hat{x}}_{k},{\text{u}}_{k},{\bar{\text{w}}}_{k}+h{\text{s}}_{\text{w},j}\right)-{\text{f}}_{i}\left({\hat{x}}_{k},{\text{u}}_{k},{\bar{w}}_{k}-h{\text{s}}_{w,j}\right)\right\}$$

(9) $${\text{S}}_{\text{x}\hat{\text{x}},k}^{\left(2\right)}=\frac{\sqrt{h^{2}-1}}{2h^{2}}\left\{{\text{f}}_{i}\left({\hat{\text{x}}}_{k}+h{\hat{s}}_{x,j},{\text{u}}_{k},{\bar{w}}_{k}\right)+{\text{f}}_{i}\left({\hat{x}}_{k}-h{\hat{s}}_{x,j},{\text{u}}_{k},{\bar{w}}_{k}\right)-2{\text{f}}_{i}\left({\hat{x}}_{k},{\text{u}}_{k},{\bar{w}}_{k}\right)\right\}$$

(10) $${\text{S}}_{\text{xw},k}^{\left(2\right)}=\frac{\sqrt{h^{2}-1}}{2h^{2}}\left\{{\text{f}}_{i}\left({\hat{x}}_{k},{\text{u}}_{k},{\bar{\text{w}}}_{k}+h{\text{s}}_{\text{w},j}\right)+{\text{f}}_{i}\left({\hat{x}}_{k},{\text{u}}_{k},{\bar{w}}_{k}-h{\text{s}}_{w,j}\right)-2{\text{f}}_{i}\left({\hat{x}}_{k},{\text{u}}_{k},{\bar{w}}_{k}\right)\right\}$$

where superscripts Equations (1) and (2) denote the first and second order divided differences, respectively, and for the Gaussian distribution, the selected interval length $h=\sqrt{3}$ [35].

Then, the a priori estimate of the state is given as

(11) $$\begin{array}{cc} {\bar{\text{x}}}_{k+1} & =\frac{h^{2}-n_{\text{x}}-n_{\text{w}}}{h^{2}}f\left({\hat{x}}_{k},u_{k},{\bar{\text{w}}}_{k}\right)\\ & +\frac{1}{2h^{2}}\overset{n_{x}}{\underset{p=1}{\text{∑}}}\left\{\text{f}\left({\hat{x}}_{k}+h{\hat{\text{s}}}_{\text{x},p},u_{k},{\bar{w}}_{k}\right)+\text{f}\left({\hat{x}}_{k}-h{\hat{s}}_{\text{x},p},u_{k},{\bar{w}}_{k}\right)\right\}\\ & +\frac{1}{2h^{2}}\overset{n_{w}}{\underset{p=1}{\text{∑}}}\left\{\text{f}\left({\hat{x}}_{k},u_{k},{\bar{w}}_{k}+hs_{\text{w},p}\right)+\text{f}\left({\hat{x}}_{k},u_{k},{\bar{w}}_{k}-hs_{\text{w},p}\right)\right\} \end{array}$$

where *n*_x_ and *n*_w_ denote the dimension of the state vector and process noise vector respectively, then the updated square root of the a priori state covariance matrix **S̄**_x_,*_k_*_+1_ is given by

(12) $${\bar{\text{S}}}_{\text{x},k+1}=HT\left(\left[\begin{array}{cccc} {\text{S}}_{\text{x}\hat{\text{x}},k}^{\left(1\right)} & {\text{S}}_{\text{xw},k}^{\left(1\right)} & {\text{S}}_{x\hat{x},k}^{\left(2\right)} & {\text{S}}_{\text{xw},k}^{\left(2\right)} \end{array}\right]\right)$$

where *HT* (**S**) denotes a Householder transformation to convert the matrix **S** into a square triangular form *HT* (**S**) *HT* (**S**)*^T^* = **SS***^T^*.

Another four matrices consisting of divided differences are defined as

(13) $${\text{S}}_{\text{y}\bar{x},k+1}^{\left(1\right)}=\frac{1}{2h}\left\{g_{i}\left({\bar{x}}_{k+1}+h{\bar{s}}_{\text{x},j},{\bar{v}}_{k+1}\right)-g_{i}\left({\bar{x}}_{k+1}-h{\bar{s}}_{\text{x},j},{\bar{v}}_{k+1}\right)\right\}$$

(14) $${\text{S}}_{\text{yv},k+1}^{\left(1\right)}=\frac{1}{2h}\left\{g_{i}\left({\bar{x}}_{k+1},{\bar{v}}_{k+1}+h{\text{s}}_{\text{v},j}\right)-g_{i}\left({\bar{x}}_{k+1},{\bar{v}}_{k+1}-h{\text{s}}_{\text{v},j}\right)\right\}$$

(15) $${\text{S}}_{\text{y}\bar{x},k+1}^{\left(1\right)}=\frac{\sqrt{h^{2}-1}}{2h^{2}}\left\{g_{i}\left({\bar{x}}_{k+1}+h{\bar{s}}_{\text{x},j},{\bar{v}}_{k+1}\right)-g_{i}\left({\bar{x}}_{k+1}-h{\bar{s}}_{\text{x},j},{\bar{v}}_{k+1}\right)-2g_{i}\left({\bar{x}}_{k+1},{\bar{v}}_{k+1}\right)\right\}$$

(16) $${\text{S}}_{\text{yv},k+1}^{\left(1\right)}=\frac{\sqrt{h^{2}-1}}{2h^{2}}\left\{g_{i}\left({\bar{x}}_{k+1},{\bar{v}}_{k+1}+h{\text{s}}_{\text{v},j}\right)-g_{i}\left({\bar{x}}_{k+1},{\bar{v}}_{k+1}-h{\text{s}}_{\text{v},j}\right)-2g_{i}\left({\bar{x}}_{k+1},{\bar{v}}_{k+1}\right)\right\}$$

The a priori estimate of the output and the square root of its covariance matrix is calculated in a similar fashion as for the states

(17) $$\begin{array}{cc} {\bar{y}}_{k+1} & =\frac{h^{2}-n_{\text{x}}-n_{\text{v}}}{h^{2}}\text{h}\left({\bar{\text{x}}}_{k},{\bar{\text{v}}}_{k}\right)\\ & +\frac{1}{2h^{2}}\overset{n_{x}}{\underset{p=1}{\text{∑}}}\left\{\text{h}\left({\bar{x}}_{k+1}+h{\bar{\text{s}}}_{\text{x},p},{\bar{v}}_{k+1}\right)+\text{h}\left({\bar{x}}_{k+1}-h{\bar{\text{s}}}_{\text{x},p},{\bar{v}}_{k+1}\right)\right\}\\ & +\frac{1}{2h^{2}}\overset{n_{v}}{\underset{p=1}{\text{∑}}}\left\{\text{h}\left({\bar{x}}_{k+1},{\bar{v}}_{k+1}+hs_{\text{v},p}\right)+\text{h}\left({\bar{x}}_{k+1},{\bar{v}}_{k+1}-hs_{\text{v},p}\right)\right\} \end{array}$$

(18) $${\text{S}}_{\text{y},k+1}=HT\left(\left[\begin{array}{cccc} {\text{S}}_{\text{y}\hat{x},k+1}^{\left(1\right)} & {\text{S}}_{\text{yv},k+1}^{\left(1\right)} & {\text{S}}_{\text{y}\hat{x},k+1}^{\left(2\right)} & {\text{S}}_{\text{yv},k+1}^{\left(2\right)} \end{array}\right]\right)$$

The gain matrix K*_k_*_+1_ is given by

(19) $${\text{K}}_{k+1}={\bar{\text{S}}}_{\text{x},k+1}{\left({\bar{\text{S}}}_{\text{y}\bar{\text{x}},k+1}^{\left(1\right)}\right)}^{T}{\left(S_{y,k+1}{\left(S_{y,k+1}\right)}^{T}\right)}^{-1}$$

Finally, the a posteriori update of the state vector and the square root of the a posteriori state covariance matrix is given by

(20) $${\hat{x}}_{k+1}={\bar{x}}_{k+1}+{\text{K}}_{k+1}\left({\text{y}}_{k+1}-{\bar{y}}_{k+1}\right)$$

(21) $${\hat{S}}_{x,k+1}=HT\left(\left[\begin{array}{cccc} {\bar{S}}_{\text{x},k+1}-K_{k}{\bar{S}}_{\text{x},k+1}^{\left(1\right)} & K_{k}{\bar{S}}_{\text{yv},k+1}^{\left(1\right)} & K_{k}{\bar{S}}_{\text{y}\hat{\text{x}},k+1}^{\left(2\right)} & K_{k}{\bar{S}}_{\text{yv},k+1}^{\left(2\right)} \end{array}\right]\right)$$

### 3.2. Modification of Measurement Update Using Huber's Technique

The Huber method [28,29] is a recursive algorithm, in which the actual measurements and the state correction attained takes the form of a linear regression problem between the predicted state and the observed quantity. Using this technique, some robust filtering approaches [26,32,36–38] have been developed and successfully applied to elliptical orbit design and tracking problems. To apply this method in the DDF, it is first required to recast the measurement update as a regression problem between the observed quantity and the state prediction. If the true value of the state is written as **x***_k_* and the state prediction error is written as **δ***_k_* =**x***_k_* − **x̄***_k_*, and then the linear regression problem has the form [30]

(22) $$\left\{\begin{array}{c} {\text{y}}_{k}\\ {\bar{x}}_{k} \end{array}\right\}=\left[\begin{array}{c} {\text{H}}_{k}\\ \text{I} \end{array}\right]x_{k}+\left\{\begin{array}{c} {\text{v}}_{k}\\ -\delta_{k} \end{array}\right\}$$

By definition of the quantities

(23) $${\text{z}}_{k}=\Lambda_{k}^{-1}\left\{\begin{array}{c} {\text{y}}_{k}-{\bar{y}}_{k}+{\text{H}}_{k}{\bar{x}}_{k}\\ {\bar{x}}_{k} \end{array}\right\}$$

(24) $${\text{M}}_{k}=\Lambda_{k}^{-1}\left\{\begin{array}{c} {\text{H}}_{k}\\ \text{I} \end{array}\right\}$$

(25) $$\xi_{k}=\Lambda_{k}^{-1}\left\{\begin{array}{c} {\text{w}}_{k}\\ -\delta_{k} \end{array}\right\}$$

(26) $$\Lambda_{k}^{-1}=\left[\begin{array}{cc} {\text{S}}_{\text{w},k} & 0\\ 0 & {\bar{\text{S}}}_{\text{x},k} \end{array}\right]$$

the linear regression problem is transformed to

(27) $${\text{z}}_{k}={\text{M}}_{k}{\text{x}}_{k}+\xi_{k}$$

In this transformed regression problem, the covariance of **ξ***_k_* is simply the identity matrix, as can be seen from expanding the expectation $E\left(\xi_{k}\xi_{k}^{T}\right)$.

The Huber filter measurement update can be solved by minimizing the cost function

(28) $$J\left({\text{x}}_{k}\right)=\sum_{i=1}^{l}\rho \left(\zeta_{i}\right)$$

where **ξ***_i_* refers to pthe *i* th component of the residual vector **ξ***_k_* =**M***_k_***x***_k_* −**z***_k_*, *l* is the dimension of **ξ**, and the score function *ρ* is defined as

(29) $$\rho \left(\zeta_{i}\right)=\{\begin{array}{ll} \frac{1}{2}\zeta_{i}^{2} & \text{for}\left|\zeta_{i}\right|<\gamma \\ \gamma \left|\zeta_{i}\right|-\frac{1}{2}\gamma^{2} & \text{for}\left|\zeta_{i}\right|\geq \gamma \end{array}$$

where *γ* is a tuning parameter. When applied to contaminated Gaussian or outlier measurements, the estimates minimize the maximum asymptotic estimation variance and have desirable robustness properties.

The solution of the Huber regression problem is determined from the derivative of the cost function

(30) $$\frac{\partial J\left({\text{x}}_{k}\right)}{\partial {\text{x}}_{k}}=\sum_{i=1}^{l}\frac{\rho \left(\zeta_{i}\right)}{\partial \zeta_{i}}\frac{\partial \zeta_{i}}{\partial {\text{x}}_{k}}=\sum_{i=1}^{l}\phi \left(\zeta_{i}\right)\frac{\partial \zeta_{i}}{\partial {\text{x}}_{k}}=0$$

Please note that

(31) $$\phi \left(\zeta_{i}\right)=\rho^{\prime }\left(\zeta_{i}\right)=\{\begin{array}{ll} \left|\zeta_{i}\right| & \left|\zeta_{i}\right|<\gamma \\ \gamma \mathrm{sgn}\left(\zeta_{i}\right) & \left|\zeta_{i}\right|\geq \gamma \end{array}$$

The solution of Equation (30) is typically obtained by using the iteratively reweighted approach developed by Beaton and Tukey in [39]. By defining the function *ψ* (*ξ_i_*) = *φ*(*ξ_i_*)/ *ξ_i_* and the matrix **ψ** = diag [*ψ*(*ξ_i_*)], the implicit equation can be written in matrix form as

(32) $${\text{M}}_{k}^{T}\psi \left({\text{M}}_{k}{\text{x}}_{k}-{\text{z}}_{k}\right)=0$$

Equation (32) can be expanded to yield ${\text{M}}_{k}^{T}\psi {\text{M}}_{k}{\text{x}}_{k}={\text{M}}_{k}^{T}\psi z_{k}$, which can be solved for **x***_k_* to give ${\text{x}}_{k}={\left({\text{M}}_{k}^{T}\psi {\text{M}}_{k}\right)}^{-1}{\text{M}}_{k}^{T}\psi {\text{z}}_{k}$, because the matrix **ψ** depends on the residuals **ξ***_i_*, and hence on **x***_k_*, the iterative solution can be expressed as

(33) $${\text{x}}_{k}^{\left(j+1\right)}={\left({\text{M}}_{k}^{T}\psi^{\left(j\right)}{\text{M}}_{k}\right)}^{-1}{\text{M}}_{k}^{T}\psi^{\left(j\right)}{\text{z}}_{k}$$

where the superscript (*j*) refers to the iteration index. The method can be initialized by using the least-squares solution ${\text{x}}_{k}^{\left(0\right)}={\left({\text{M}}_{k}^{T}{\text{M}}_{k}\right)}^{-1}{\text{M}}_{k}^{T}{\text{z}}_{k}$, corresponding to **ψ** = **I**. The converged value from the iterative procedure is taken as the state estimate **x̂***_k_*, and the iteratively reweighted approach converges due to no increasing functions.

Finally, the state estimate error covariance matrix and the corresponding Cholesky factor are computed from Equations Equations (34) and (35) respectively, using the final value of **ψ** corresponding to the converged state estimate.

(34) $${\hat{P}}_{k}={\left({\text{M}}_{k}^{T}\psi {\text{M}}_{k}\right)}^{-1}$$

(35) $${\hat{S}}_{x,k}=\text{chol}\left({\hat{P}}_{k}\right)$$

Notice that as *γ* → ∞, the matrix **ψ → I**, the Huber filtering problem reduces to the least-squares estimator and the Huber-based DDF measurements update is identical to the classical DDF solution. Also note that as *γ* → 0, the problem reduces to the absolute value estimator. This blend of estimation techniques provides the HDDF estimator robustness against deviations from Gaussian distributed random measurements errors [26,36,40]. This robustness arises from the **ψ** matrix and that all residuals are not weighted equally. In particular, the large residuals are down-weighted in the iterative solution technique by the inverse of the magnitude of the residual [41–44].

### 3.3. Robust Huber-Based Iterated Divided Difference Filter

As discussed previously, the IDDF avoids truncated error of high order as DDF, and well satisfies the situation of weak observability and large initial error, so it increases the filtering accuracy to a certain extent. However, its performance heavily depends on the accuracy of the measurements; the contaminated measurements with outliers will degrade the performance of IDDF greatly. In contrast, the Huber's M-estimation methodology is robust to the outliers and ensures the accuracy of the estimation. In view of the IDDF and Huber's M-estimation methodology, the robust HIDDF is put forward to improve the filter's performance in this work.

**Figure 1.**
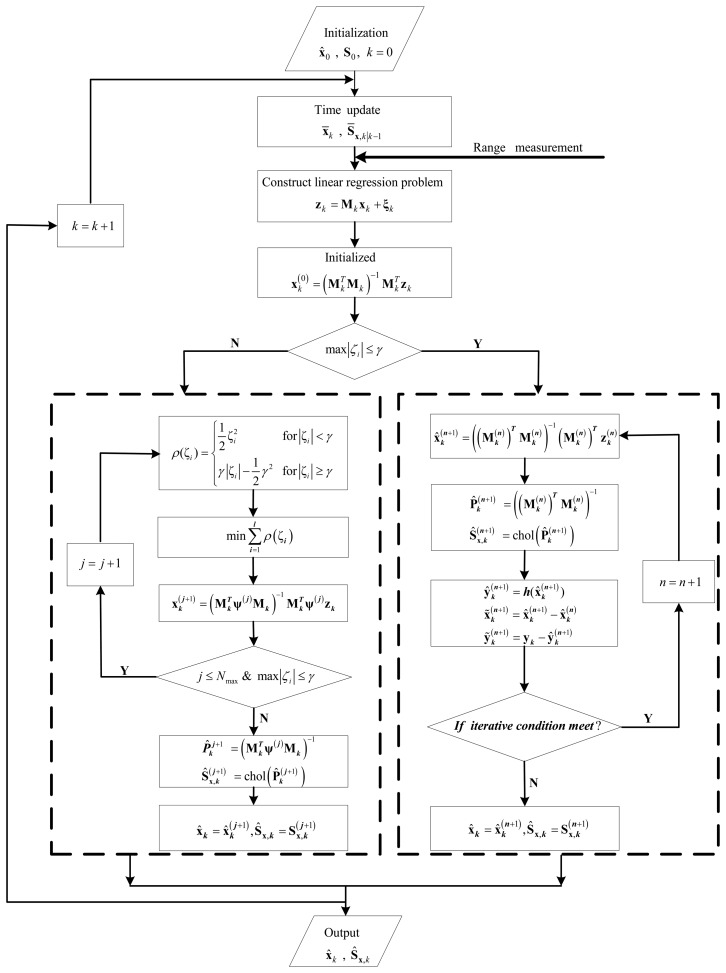
Flow of HIDDF algorithm.

Figure 1 shows the flow of the HIDDF algorithm. The switch function max |ξ*_i_*| is the maximum component of the residual vector **ξ** = **M***_k_***x***_k_* − **z***_k_*, and *γ* is a given error threshold. When max |ξ*_i_*| ≤ *γ*, the estimated state reaches the expected accuracy demand and the Huber-based measurement update is identical to the classical DDF solution because of **ψ → I**. In this situation, the simplified iterated method is adopted to improve the performance further with the up-to-date estimated information that is used as the initial estimate for each iteration process. The superscript (*n*) denotes the iteration number of the measurement update and the iteration procedure continues until the criterion function to iteration termination is satisfied as follows

(36) $${\left({\tilde{x}}_{k}^{\left(n+1\right)}\right)}^{T}{\left({\text{P}}_{k}^{\left(n+1\right)}\right)}^{-1}{\tilde{x}}_{k}^{\left(n+1\right)}+{\left({\tilde{y}}_{k}^{\left(n+1\right)}\right)}^{T}{\text{R}}_{k}^{-1}{\tilde{y}}_{k}^{\left(n+1\right)}>{\left({\tilde{y}}_{k}^{\left(n\right)}\right)}^{T}{\text{R}}_{k}^{-1}{\tilde{y}}_{k}^{\left(n\right)}$$

or the iteration number is too large (*n* > *N*_max_) [18,23]. On the contrary, when max |ξ*_i_*| > *γ*, the filter has a divergent trend because of the contaminated measurements with outliers. In this case, to increase the filtering robustness, instead of iterating the measurement update, Huber's M-estimation methodology is used.)

The sensitivity of the robustness to the contaminated measurements depends on the error threshold *γ*. The smaller the threshold value that is set, the more sensitive the robustness became to the contaminated measurements and, contrarily, the weaker the robustness. It is noteworthy that the error threshold *γ* is not constant here. For the robust Huber methodology, a small threshold value is usually used to identify the contaminated measurement noise. However, it is not effective in the conditions of a large initial error, because the divergent estimate caused by the initial error will be attributed to measurement noise. Apparently, the succedent robust methodology in this situation will exacerbate the filtering performance in comparison to the standard filtering algorithm. In addition, the convergence speed will not be increased due to the range measurements not being able to be used adequately. In this robust method, the threshold *γ* was set to a large value during the first few measurement update processes in order to ensure a fast convergence speed in large initial error used the iterated process. Subsequently, a small threshold value was used to make sure the system had a good robustness to outlier measurements.

#### Remark

In virtue of the proving method of the advantage in weak observability and large initial errors of iterated Unscented Kalman Filter in [22], we can prove the advantage in tracking accuracy and convergence speed of the IDDF algorithm in AUV cooperative localization. The robustness of the Huber-based DDF method to the contaminated outlier measurement has been proved in [36,40]. The proposed HIDDF filter switches between the IDDF and the Huber-based DDF through switch mode function and error threshold, which does not affect the character of the two methods and combines the advantages of both, so the HIDDF method does not only achieve a more accurate position estimate in weak observability and faster convergence speed in large initial error, but also an improved robustness to the outlier measurements.

## 4. Lake-Water Field Trials

**Figure 2.**
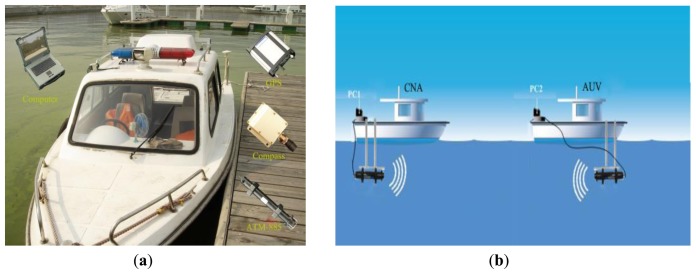
(**a**) Vessel used in our work and the on-board equipment; (**b**) Underwater acoustic communication network.

To explore the effectiveness of the proposed algorithm, we applied it to outfield experiments through simulation using data collected in field testing. Note that all the tests are executed on Matlab R2011a installed on a computer with Intel(R) Core(TM) 2 Duo T5470@1.60 GHz CPU and 2 GB memory. The test was conducted in October 2012 in Lake Thai which ranges for the most part between seven and sixteen feet deep. In these experiments, two survey vessels, as shown in Figure 2a, were used instead in our work and only a single surface leader known as CNA was used to support the one as AUV. As shown in Figure 2b that illustrates the schematic diagram of test, any vehicle outfitted with an acoustic modem ATM-885, meaning that anyone can broadcast information to another. In addition, a magnetic compass providing the course, and GPS enabling the collection of the true position were also equipped on each one. Furthermore, a DVL was also equipped on the AUV to acquire velocity information in body coordinate. The performance parameters of the above sensors are listed in Table 1. During these field trials the DR position was updated in a frequency of 1 Hz with covariance **Q***_k_* = *diag* [(0.05 m/s)^2^ (0.05 m/s)^2^ (0.05 rad)^2^]. Two-way acoustic communication between the AUV and the CNA was used and the acoustic data packets were sent from the CNA to the AUV every 15 s.

### 4.1. Cooperative Localization Using a Static Surface Leader

**Figure 3.**
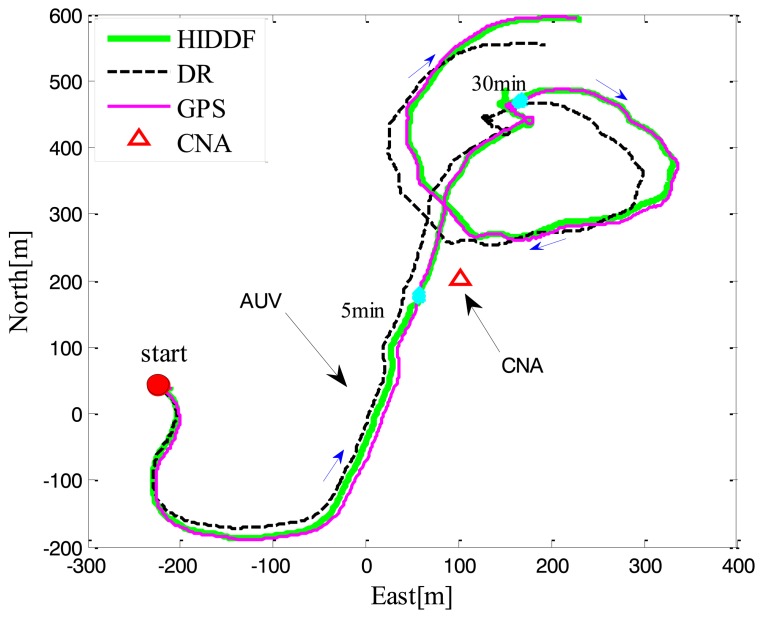
Paths taken by the AUV and CNA during Experiment 1; the static CNA is shown as red triangle, the blue arrow represents the direction of the AUV movement.

In this experiment, a single static surface leader was used to support the AUV. As shown in Figure 3, in which the path taken by the vehicles is illustrated, the CNA as leader is static while the AUV is in motion. The test lasted 40 min and in total the AUV traveled 2100 m with an average velocity of 0.84 m/s. From the localization trajectories of the DR and HIDDF, it is can be seen that the DR trajectory (black dashed line) gradually diverges from the ground truth (pink solid line), which is because the DR only employs the inertial measurements and velocities. In contrast, the estimated trajectory of the HIDDF (green solid line) has bounded localization errors thanks to the use of range measurements from the CNA.

### 4.2. Cooperative Localization Using a Surface Mobile Vehicle

**Figure 4.**
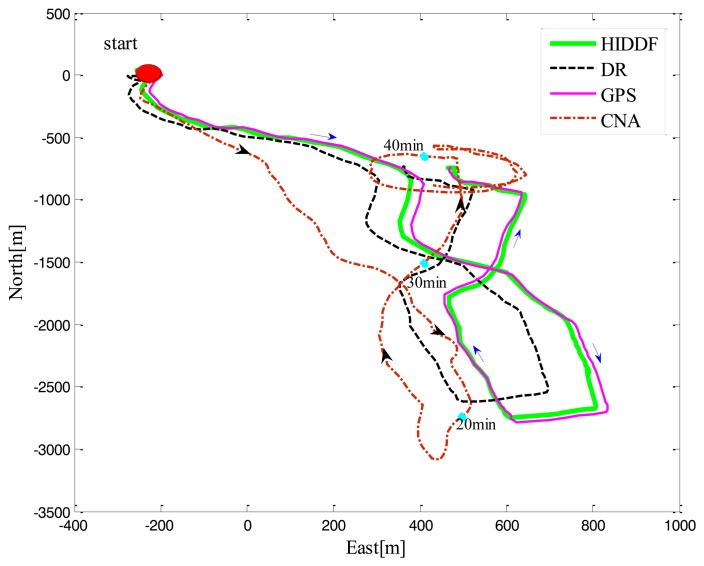
Paths taken by the AUV and CNA during Experiment 2. The red dash line represents the CNA movement trace and the black and blue arrows represent the direction of the CNA and AUV movement respectively.

In this experiment, the AUV carried out a figure-eight track, covering approximately 5240 m in a total period of 50 min while the surface mobile vehicle CNA maintained a supporting pattern, consisting of a parallel movement initially for about 35 min and then a circular sailing around the AUV for about 15 min, as shown in Figure 4. Note that the average velocity of AUV was 1.74 m/s and that of CNA was 2.45 m/s. From the localization trajectories of DR and HIDDF, it also can be seen that the HIDDF method is far superior to the DR.

### 4.3. Comparison of Filtering Algorithms

**Figure 5.**
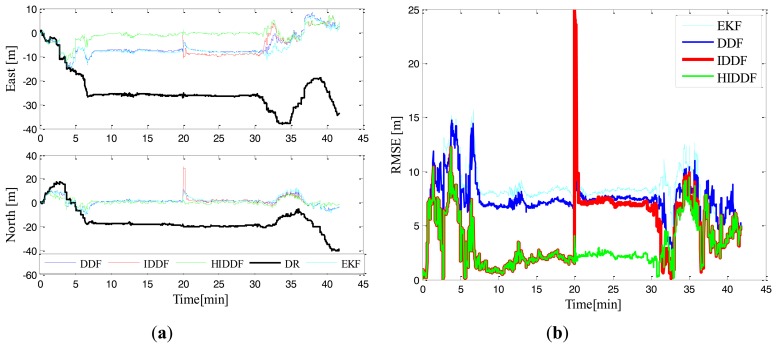
Simulation results in Experiment 1. (**a**) Position errors of different methods; (**b**) Position errors of EKF, DDF, IDDF and HIDDF.

To demonstrate the effectiveness of the proposed robust cooperative method, a comparison between the DDF, IDDF and HIDDF estimators were performed. The localization results of DR, EKF, DDF, IDDF and HIDDF in the scenario with a single static surface leader are shown in Figure 5 and Table 2. It can be seen from Figure 5a that the DR performs a larger position error than the cooperative scheme, which is because the DR only employs the compass measurements and velocities. By using the range measurements from the CNA, the EKF has bounded localization errors; however, it is less accurate than the DDF, which can be seen clearly from the localization errors in Figure 5b. This is mainly because the linearization of a nonlinear system in the EKF degrades the accuracy of estimation, while the DDF can handle the nonlinearity more precisely than EKF. As shown in Figure 5b, the IDDF and HIDDF perform similarly in 20 min before and better than the conventional DDF in position estimate accuracy. One of the reasons for this is that the conventional DDF method only utilizes range observation in the update once and can therefore not use the range information sufficiently, resulting in the ineffective estimate of the position; whereas the iterated method (IDDF and HIDDF) in our work can make use of the range measurements sufficiently by using an iterative measurement update. Furthermore, the linearization of a nonlinear system in the DDF degrades the accuracy of estimation, while the iterative method can handle the nonlinearity more precisely than DDF by iteratively solving the optimization problem.

Because of the high-quality acoustic communication and the absence of range measurement outliers in tests, we simulated a typical outlier measurement by setting the range measurement obtained by the AUV at the twentieth minute from 262.63 m to 302 m; all subsequent range measurements were unchanged. It clearly can be seen from Figure 4 that the HIDDF is superior to the IDDF in the robustness to the outlier measurement. Upon receipt of the outlier measurement in the twentieth minute, there is almost no influence on the position estimate of the HIDDF. However, the error of the position estimate “jumps” for the conventional EKF, DDF and IDDF methods, most significantly for the IDDF algorithm, and is subsequently slowly converged. This is due to the outlier measurement making the conventional DDF divergent and the iterated use of the measurement in IDDF subsequently enlarges this influence. The proposed HIDDF method, however, has good robustness to the outlier measurement and improves the stability of the IDDF method in conditions of contaminated measurement noise.

**Figure 6.**
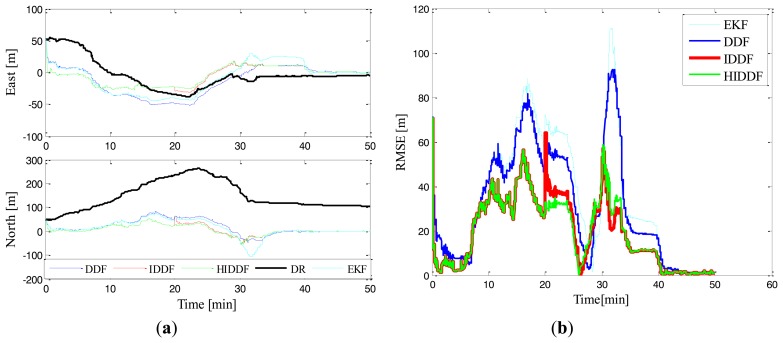
Simulation results in Experiment 2. (**a**) Position errors of different methods; (**b**) Position errors of EKF, DDF, IDDF and HIDDF.

Figure 6 presents the localization errors of DR, EKF, DDF, IDDF and HIDDF in the context of a single surface mobile vehicle as leader. As shown in Figure 6, it also can be seen that the DR position is unbounded, the EKF is less accurate than the DDF in cooperative scheme and the estimated position of AUV by using conventional DDF is still inferior to the IDDF and HIDDF methods. This is particularly important for the segments of the mission in which poor relative vehicle motion results in poor AUV observability during the first 40 min. As for the last 10 min, because of the circular movement of the CNA that results in strong AUV observability, the conventional DDF converged fast and performed as well as the IDDF and HIDDF. It can be illustrated that the iterative method can suit AUV localization well even with poor observability. In this experiment, we also simulated a typical outlier measurement by setting the range measurement obtained by the AUV at the twentieth minute from 436.91 m to 476.91 m and the corresponding localization errors also verifies that the performance of IDDF in terms of robustness to outlier measurement is worse than that of HIDDF. The reasons why HIDDF is superior to IDDF and conventional DDF are similar to the ones described in the static leader scenario.

Table 2 shows the simulation results of different methods according to 0–20 min, 20–30 min. By comparing the mean position errors before and after the twentieth minute, we can see that the IDDF and HIDDF achieves a similar precision and better than the other methods before twentieth minute, which is because the iterative measurement update in IDDF and HIDDF makes use of the range measurements sufficiently. However, during the 20∼30 min, the HIDDF achieves a much better performance than the IDDF. This is because the outlier measurements at the twentieth minute make the IDDF divergent and subsequently slowly converge as shown in Figures 5b and 6b; the HIDDF, however, is robust to the outlier measurements and consistently performs better.

**Figure 7.**
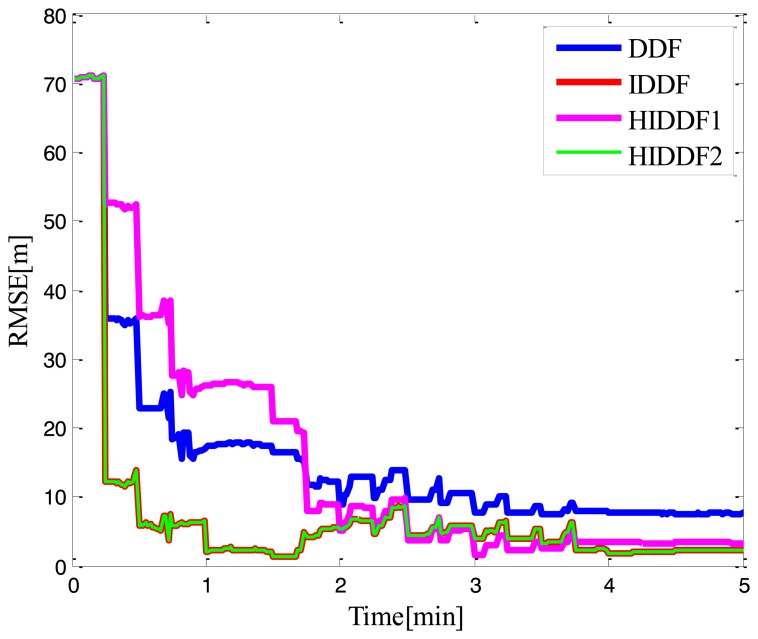
Comparison of convergence speed in large initial error; the HIDDF1 represents the HIDDF method with constant threshold value and the HIDDF2 represents the proposed HIDDF method with a variable threshold value.

To evaluate the performance of convergence speed in conditions of large initial error, we set the initial position state to **x̂**_0_ = [50m 50m] in Experiment 2. It is also seen from Figure 6b that both the IDDF and HIDDF have a faster convergence speed than the conventional EKF and DDF method, because these two filters iterated the measurement update using the accurate range measurements. As discussed in Section 3.3, in this work, the threshold *γ* was set to 50 (the value is not fixed, it is possible as long as it is large enough to make the robustness insensitive to the measurements) before the first five measurement updates are processed to ensure a fast convergence speed. Because of the weak observability for AUV localization with a single leader, the estimated position usually has a large uncertainty, and so a larger threshold value of 15 than in [30,34] (the threshold value was set according to the experiment) is set for subsequent time to reduce the sensitivity to the residual vector. To further validate the method of the threshold choice in this work, a comparison between the proposed method and a simple method in which the threshold γ was set to a constant value 15, were also performed. As shown in Figure 7, the HIDDF with constant threshold value has a lower convergence speed than the proposed method and even worse accuracy of position estimate than the conventional DDF, which is because the range measurements were thought to be contaminated for the small threshold value due to the large initial error and then the robust methodology is used for it. As for the HIDDF2, due to the larger threshold value used during the first few measurement update process, the range measurements were thought accurate and iterated used like the traditional IDDF method. The convergence speed of the HIDDF2 is thus similarto the IDDF. By these numerical evaluations, it is verified that the proposed HIDDF method not only achieves accurate localization estimation and faster convergence speed in weak observability and large initial error, but also is more robust to outliers of acoustic range measurements than conventional IDDF and DDF in the context of single beacon; it is therefore more suitable for the single leader-based localization.

## 5. Conclusions

This paper has described a new robust Huber-based HIDDF algorithm for AUV cooperative localization and its validation through simulation using data collected in field testing. The algorithm is particularly well suited for AUV cooperative localization, in which weak observability, large initial error and outlier measurements are unavoidable. By integrating the robust Huber's M-estimation methodology into the IDDF algorithm, the HIDDF achieves lower position error in weak observability and faster convergence speed in large initial error and, more importantly, robustness to outlier measurements, thereby avoiding sharp divergence of estimation error caused by iterated update using the outlier measurements and improving the stability of the iterated method. The presented method can also be extended to other problems of nonlinear filtering.

Our future work will focus on implementing the algorithm in a larger network, in which a set of heterogeneous vehicles are continuously submerged with only a single vehicle occasionally surfacing to access GPS measurements.

## Figures and Tables

**Table 1.** The performance parameters of equipment used in experiment.

**Sensors**	**Metric**	**Parameters**
Compass	Random noise	2°
GPS	Position accuracy	1.8 m (CEP)
	Data update rate	10 Hz
DVL	Working range	−150 m/s–200 m/s
	Measurement accuracy	0.1%
ATM-885	Working range	Up to 8000 m
	Error rate	Less than 10^−7^

**Table 2.** Localization errors of different methods.

	**Exp.1**					**Exp.2**				
	
	**DR**	**EKF**	**DDF**	**IDDF**	**HIDDF**	**DR**	**EKF**	**DDF**	**IDDF**	**HIDDF**
0~20 min	26.07	8.76	8.36	3.46	3.44	141.54	40.84	38.02	22.96	22.93
20~30 min	32.79	8.58	8.34	6.94	2.18	236.93	42.88	36.01	27.51	23.60
